# Genomic Sequencing To Identify Potential Causative Mutation(s) of Neurospora crassa *col-4*

**DOI:** 10.1128/MRA.01009-19

**Published:** 2020-01-09

**Authors:** Thomas A. Randall

**Affiliations:** a Ronin Genetics, Durham, North Carolina, USA; University of California, Riverside

## Abstract

In many cases, genes for commonly used genetic markers in model organisms have not been identified; therefore, it is of interest to identify the causative genes. Whole-genome sequencing was used to identify potential causative mutations for a *col-4* allele of Neurospora crassa.

## ANNOUNCEMENT

The low cost of genomic sequencing has made it feasible to bypass traditional genetics-based methods of gene identification and to approach this directly by sequencing the entire genome of interest. This has become very common in model systems with small genomes but is now being applied on a much larger scale to larger genomes (specifically, human genomes) in various cancer genome projects. The *col-4* locus of Neurospora crassa, on linkage group (LG) IVR, has a colonial phenotype and has long been used in genetic studies. A single allele, Y152M43;30(r) 70007, was isolated in 1949 (1) and was originally used to mimic colonial growth before the advent of sorbose-containing media. The gene for *col-4* has not been characterized, and finding a candidate gene (or genes) for *col-4* is the major goal of this project.

N. crassa strains FGSC 3017 (mating type *a*, *his-2*;*mtr col-4*) and FGSC 2489 (mating type *A*) were obtained from the Fungal Genetics Stock Center (2). Standard media with appropriate supplements were used (3). N. crassa strain 3017 was crossed to N. crassa strain 74A, and a single spore progeny (TR1) (mating type *a*, *his-2*;*mtr col-4*) was used for further analysis; *mtr* (encoding a neutral amino acid permease), on LG IV ∼1 map unit from *col-4*, has been well characterized, but this *mtr* allele has not. Similarly, the *his-2* allele (encoding ATP phosphoribosylpyrophosphate pyrophosphorylase) on LG IR has not been characterized. DNA was isolated from N. crassa TR1 (4), and 50 μg was shipped to Operon MWG (Huntsville, AL) for sequencing. DNA was nebulized to 500 bp, one lane of 100-bp paired-end sequence was generated with the Illumina HiSeq 2000 platform using TruSeq chemistry, and 146,243,715 paired-end sequence reads in fastq format were obtained (approximately 600× coverage of N. crassa). FastQC (https://www.bioinformatics.babraham.ac.uk/projects/fastqc) was used for quality control. No quality trimming or adapter trimming was found to be necessary. For single-nucleotide polymorphism (SNP)/indel identification, two separate subsets of 20 million reads from each paired-end fastq file were loaded onto the Galaxy analysis platform (https://usegalaxy.org) (5). The N. crassa reference sequences (build 10) for LG I and LG IV in fasta format were downloaded from the Broad Institute website (https://www.broadinstitute.org/fungal-genome-initiative) and loaded onto the Galaxy platform. Reads were aligned to each reference sequence with BWA 0.6.0 (6), and SNPs and indels were determined with the mpileup function of SAMtools 1.9 (7), by manual inspection. Three candidates for *col-4* were identified, namely, an A-to-G transition 100 bp upstream of locus NCU06625 (base 1842866, LG IV), a T-to-C transition upstream of locus NCU16770 (base 1882007, LG IV), and an −/A indel between locus NCU06628 and locus NCU06629 (base 1863899, LG IV); all are intergenic and are likely to be regulatory. These were the only sequence differences from the 74A reference between the *mtr* gene and the beginning of a stretch of sequence from the Mauriceville background, which demarcates the location within which *col-4* must be found. Additionally, single candidate mutations within the open reading frames of the *his-2* and *mtr* genes were identified, i.e., a G-to-C transversion causing Ala to Pro (A32P) and a C-to-G transversion causing Tyr290 to the TAG stop codon, respectively.

### Data availability.

The original sequencing data have been deposited in GenBank with BioProject accession number PRJNA548559.
